# Dietary Vitamin D3 Deficiency Increases Resistance to *Leishmania (Leishmania) amazonensis* Infection in Mice

**DOI:** 10.3389/fcimb.2019.00088

**Published:** 2019-04-09

**Authors:** Izabella Pereira da Silva Bezerra, Gabriel Oliveira-Silva, Danielle Sophia Ferreira Santos Braga, Mirian França de Mello, Juliana Elena Silveira Pratti, Joyce Carvalho Pereira, Alessandra Marcia da Fonseca-Martins, Luan Firmino-Cruz, Diogo Maciel-Oliveira, Tadeu Diniz Ramos, André Macedo Vale, Daniel Claudio Oliveira Gomes, Bartira Rossi-Bergmann, Herbert Leonel de Matos Guedes

**Affiliations:** ^1^Instituto de Biofísica Carlos Chagas Filho, Universidade Federal do Rio de Janeiro, Rio de Janeiro, Brazil; ^2^Núcleo de Doenças Infecciosas/Núcleo de Biotecnologia, Universidade Federal do Espírito Santo, Vitória, Brazil; ^3^Núcleo Multidisciplinar de Pesquisa UFRJ – Xerém em Biologia, UFRJ Campus Duque de Caxias Professor Geraldo Cidade – Universidade Federal do Rio de Janeiro, Duque de Caxias, Brazil

**Keywords:** leishmaniasis, *Leishmania amazonensis*, vitamin D, Th1, immunonutrition, IL-10

## Abstract

The leishmaniases are a group of diseases caused by *Leishmania* parasites, which have different clinical manifestations. *Leishmania (Leishmania) amazonensis* is endemic in South America and causes cutaneous leishmaniasis (CL), which can evolve into a diffuse form, characterized by an anergic immune response. Since the leishmaniases mainly affect poor populations, it is important to understand the involvement of immunonutrition, how the immune system is modulated by dietary nutrients and the effect this has on *Leishmania* infection. Vitamin D3 (VitD) is an immunonutrient obtained from diet or endogenously synthesized, which suppresses Th1 and Th17 responses by favoring T helper (Th) 2 and regulatory T cell (Treg) generation. Based on these findings, this study aims to evaluate dietary VitD influence on *L. (L.) amazonensis* experimental infection in C57BL/6 and BALB/c mice. Thus, C57BL/6 and BALB/c VitD deficient (VDD) mice were generated through dietary VitD restriction 45 days prior to infection. Both strains of VDD mice showed a more controlled lesion development compared to mice on a regular diet (Ctrl). There were no differences in serum levels of anti-*Leishmania* IgG1 and IgG2a, but there was a decrease in IgE levels in BALB/c VDD mice. Although CD4^+^ T cell number was not changed, the CD4^+^ IFN-y^+^ T cell population was increased in both absolute number and percentage in C57BL/6 and BALB/c VDD mice compared to Ctrl mice. There was also no difference in IL-4 and IL-17 production, however, there was reduction of IL-10 production in VDD mice. Together, our data indicate that VitD contributes to murine cutaneous leishmaniasis susceptibility and that the Th1 cell population may be related to the resistance of VDD mice to *L. (L.) amazonensis* infection.

## Introduction

The leishmaniases are a group of diseases caused by protozoans of the *Leishmania* genus, which are transmitted to mammalian hosts by female sandflies of the Phlebotominae family. Each year, 0.7 to 1 million new cases occur worldwide and clinical manifestations are divided into cutaneous and the lethal visceral leishmaniasis (VL) (Burza et al., 2018). Cutaneous leishmaniasis (CL) is generally characterized by a single self-healing ulcer. However, depending on the parasite species involved in infection and on the host immune status, it can evolve into a more severe manifestation (Scott and Novais, 2016). *Leishmania* (*Leishmania*) *amazonensis*, a species of the *L*. (*L*.) *mexicana* complex, is endemic in many South American countries and can cause diffuse CL, characterized by an anergic immune response and excess of parasites in multiple lesions. Moreover, cases of VL caused by *L. (L.) amazonensis* have been reported previously (Silveira et al., 2004).

Since *Leishmania* spp. amastigote forms are intracellular and mainly infect macrophages, the development of a T helper (Th) 1 immune response is desirable for disease control. Although BALB/c mice are susceptible and C57BL/6 mice are resistant to *L. (L.) major* infection, developing a Th2, and a Th1 immune response, respectively (Heinzel et al., 1989; Launois et al., 1997; Scott and Novais, 2016), this dichotomy is not observed in *L. (L.) amazonensis* infection. Most mice strains are susceptible and develop a mixed Th1/Th2 immune response with low cellular activation, as is observed in humans (Ji et al., 2002; Silveira et al., 2009; Velasquez et al., 2016).

Available treatments against leishmaniases are based on drug repositioning and have many problems such as high toxicity, high cost and development of resistant parasite strains. In addition, there is still no human vaccine approved (Rossi and Fasel, 2018). Thus, understanding disease immunobiology is necessary for the development of strategies that promote infection control.

In this context, immunonutrition is a key factor to be considered to better understand the immune system modulation by dietary nutrients, particularly in diseases that affect poor populations (McCarthy and Martindale, 2018), such as the leishmaniases.

Vitamin D3 (VitD) is an important immunonutrient that can be obtained from the diet or can be endogenously synthesized from a cholesterol precursor (7-dehydrocholesterol) through incidence of sun-UVB rays on the skin. This vitamin and its metabolites are essential for calcium homeostasis and also affect cell growth, differentiation, and function in many tissues, including the immune system (Baeke et al., 2010; Bikle, 2011).

VitD binds to the nuclear VitD receptor (VDR), which forms a complex with retinoic acid X receptor (RXR) and promotes transcription of several genes through VitD response elements (VDREs) (Pike and Meyer, 2012). VitD also induces non-genomics effects related to cell maturation, including growth factor and cytokine modulation through cytosolic pathways (Hii and Ferrante, 2016). Immune cells express VDR and they are capable of metabolizing circulating VitD to the active form 1,25-dihydroxycholecalciferol [1,25(OH)_2_D_3_], which suppresses the immune response by blocking dendritic cell (DC) differentiation and maturation, inducing a stable tolerogenic phenotype (Piemonti et al., 2000; Penna et al., 2005; Széles et al., 2009; Ferreira et al., 2012). Moreover, DCs treated with 1,25(OH)_2_D_3_ stimulate regulatory T cell (Treg) generation and impair autoreactive T cell activation (van Halteren et al., 2004; Penna et al., 2005; Unger et al., 2009). Active 1,25(OH)_2_D_3_ also induces CCR10 expression and skin-homing through the CCL27 chemokine, imprinting T cell epidermotropism (Sigmundsdottir et al., 2007). These are fundamental functions in the context of autoimmune diseases, allergies, and immunopathologies, such as leishmaniases.

A previous study demonstrated that VitD treatment has a beneficial effect on *L. (L.) mexicana* infection, controlling lesion development in BALB/c mice (Ramos-Martínez et al., 2013). In addition, VitD also plays a favorable role in canine infection by *L. (L.) infantum*, since disease progression is strongly associated with VitD deficiency in dogs (Rodriguez-Cortes et al., 2017). On the other hand, BALB/c VDR knockout mice have increased resistance to *L. (L.) major* infection (Whitcomb et al., 2012), suggesting a negative role for VitD. Overall, the role of VitD in *Leishmania* infection seems to depend on the parasite species involved. Thus, this study aims to evaluate the effect that dietary VitD has on experimental infection with *L. (L.) amazonensis* in both C57BL/6 and BALB/c mice, including its role in the development of an immune response to the parasite.

## Materials and Methods

### Mice

Female C57BL/6 and BALB/c mice of 6–8 weeks old from Instituto de Biofísica Carlos Chagas Filho, Universidade Federal do Rio de Janeiro (Brazil) were maintained in sterilized cages and received filtered water and regular pelleted food (AIN-93M, Pragsoluções, Brazil). Dietary VitD deficient (VDD) mice were subjected to a commercial diet without VitD (AIN-93M, Pragsoluções, Brazil) for 45 days before infection. The diet was maintained throughout the experiment. All procedures performed are in accordance with the “Basic Principles for Research Involving Animal Use,” approved and registered by the Ethics Committee on Animal Use in Research (CEUA/UFRJ number 157).

### Parasites

*Leishmania (L.) amazonensis* promastigotes (MHOM/BR/75) were maintained at 26°C in Minimum Essential Medium 199 (MEM 199, Cultilab, Brazil) supplemented with 10% heat inactivated fetal bovine serum (HIFBS, Cultilab, Brazil), penicillin and streptomycin (100 U/mL and 100 μg/mL, respectively, Stemcell Technologies, USA) and hemin (5 μg/mL, Sigma-Aldrich, USA). To ensure infectivity, parasites were used at most until the fourth culture passage, after which, parasites were reisolated from pre-infected BALB/c mice.

### Infection

Culture promastigotes in the stationary growth phase were used for infection. Parasites were washed with PBS by centrifugation at 1500 g. Mice were subcutaneously injected into the hind footpad with 2 × 10^5^ *L. (L.) amazonensis* promastigotes in a volume of 20 μL. Lesion development was monitored by measurement with a caliper (No. 7301, Mitutoyo, Japan) once every 7 days for 92 (C57BL/6 mice) or 99 (BALB/c mice) days.

### Parasite Load

Parasite loads were evaluated by limiting dilution assay as previously described (Torres-Santos et al., 1999). Briefly, on day 92 (C57BL/6 mice) or 99 (BALB/c mice) post-infection, footpads were individually homogenized (2 mL/footpad), and diluted 1:100 (only BALB/c mice samples) in MEM 199. Homogenates were serially diluted 1:4 in microplates for 16 dilutions (final volume of 200 μL/well), performed in triplicate for each sample, and incubated at 26°C for 14 days. The original number of amastigotes per footpad was calculated taking as reference the last dilution in which promastigotes were observed under optical microscope, theoretically equivalent to a single amastigote.

### Cytokines

IL-4, IL-17, and IL-10 were measured in supernatants of lesion homogenates by Enzyme-Linked Immunosorbent Assay (ELISA). Cytokine concentrations were determined from standard curves using recombinant cytokines and murine antibodies, according to manufacturer's instructions (BD Systems, USA).

### Antibodies

On day 92 (C57BL/6 mice) or 99 (BALB/c mice) post-infection, blood samples were collected, and after standing at room temperature for 2 h, then centrifuged at 2000 g to obtain serum. Seric IgG1, IgG2a, IgA, and IgE antibodies specific for *L. (L.) amazonensis* promastigote antigens (LaAg) were quantified by ELISA. LaAg was prepared as described before (Pratti et al., 2016). Briefly, plates were pretreated with LaAg (1 μg/well) and incubated overnight at 4°C. Samples were diluted 250x for evaluation and biotinylated antibodies were used for detection (Goat Anti-Mouse IgG1-UNLB: Cat. No. 1071-01, Goat Anti-Mouse IgG2a-UNLB: Cat. No. 1101-01, Goat Anti-Mouse IgE-UNLB: Cat. No. 1110-05, Goat Anti-Mouse IgA-UNLB: Cat. No. 1040-05, SouthernBiotec) according to the manufacturer's recommendations.

### Flow Cytometry

On day 92 (C57BL/6 mice) or 99 (BALB/c mice) post-infection, popliteal lesion-draining lymph nodes homogenates were prepared, cellularity was performed by manual counting on a Neubauer chamber and single cell suspensions were incubated with anti-CD3 (Peridinin Chlorophyll Protein Complex; Percp-conjugated), anti-CD4 [R-phycoerythrin PE and the cyanine dye Cy7 combined; PE-Cy7-conjugated], and anti-IFN-y (Allophycocyanin; APC-conjugated) monoclonal antibodies according to manufacturer's instructions from eBioscience. The analyzes were performed in the software Summit. Gate strategy is demonstrated in Supplementary Figure 5.

### Statistical Analysis

Results were analyzed using GraphPad PRISM® version 6.0 software. Student *t*-test was used. For lesion development kinetics a Two-way ANOVA with Bonferroni post-test was used. Results are expressed as mean ± standard deviation (SD) and the differences between means with *P* < 0.05 are considered significant. All data are representative of three independent experiments.

## Results

### VDD Mice Are More Resistant to *L. (L.) amazonensis* Infection

**Figure 1:**
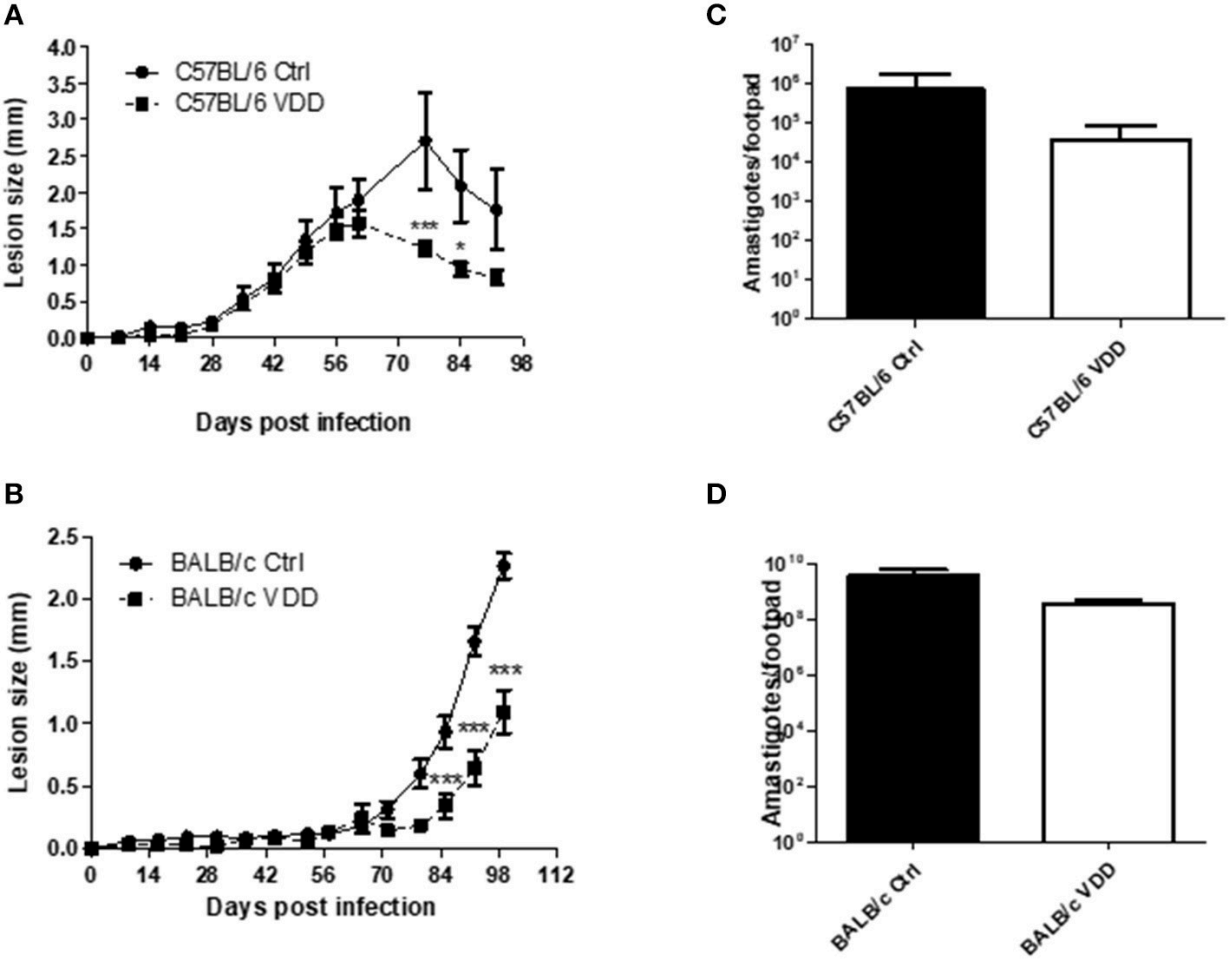
VDD mice are more resistant to *L. (L.) amazonensis* infection. C57BL/6 and BALB/c mice normally fed (Ctrl) or on a Vitamin D-deficient diet (VDD) were subcutaneously infected in the footpad with 2 × 10^5^ *L. (L.) amazonensis* promastigotes and lesion development was followed weekly **(A,B)**. On day 92 (C57BL/6) or 99 (BALB/c) post-infection, parasite loads in the infection site were evaluated by limiting dilution assay **(C,D)**. The data (means ± SD; *n* = 5 ^***^P < 0.0001; ^*^P < 0.05) are representative of three independent experiments producing the same result profile.

The development of *L. (L.) amazonensis* infection was evaluated in partially susceptible C57BL/6 and susceptible BALB/c mice submitted to dietary VitD restriction (VDD) or fed a regular diet (Ctrl). Both C57BL/6 and BALB/c VDD mice showed a more controlled lesion development compared to those on the regular diet (Figures 1A,B) when mice were infected with 2 × 10^5^ promastigotes. In C57BL/6 VDD the lesions began to resolve by day 60, whereas in the Ctrl mice this did not occur until day 75. In BALB/c mice, the lesions continued to develop until the endpoint of the experiment, with BALB/c VDD mice showing a significantly smaller lesion over this time. There was no significant difference in the parasite loads at the infection site of the C57BL/6 VDD compared to C57BL/6 ctrl (day 92) or BALB/c VDD compared to BALB/c ctrl (day 99) (Figures 1C,D). We also evaluated parasite load in lymph nodes, and no difference was observed between the infected Ctrl and VDD mice for either C57BL/6 and BALB/c mice (Supplementary Figure 1). It is important to note that dietary VitD restriction did not interfere in the appearance or body weight of the mice (Supplementary Figure 2). Moreover, when a high challenge model of infection with 2 × 10^6^ promastigotes was used, no difference was observed in lesion size between the Ctrl and VDD mice for each mouse strain (Supplementary Figure 3). Based on that, subsequent experiments were performed using an infection dose of 2 × 10^5^ parasites. However, as we had already observed that the lesion resolution was more accelerated in the C57BL/6 VDD mice (Figure 1), we further evaluated the infection profile of these mice for 149 days to assess whether there was complete resolution of the lesion. In the beginning of chronic phase there was no difference between the VDD and Ctrl mice, however, later in the chronic phase, VDD mice presented significantly smaller lesions in comparison to the Ctrl mice (Supplementary Figure 4) indicating a reduction of lesion size at both the peak of infection and in late chronic phase. However, again, there was no significant difference in the parasite loads at this late chronic phase time-points.

### Increase in CD4^+^ IFN-γ^+^ T Cell Population in the Lesion-Draining Lymph Nodes of VDD Mice

**Figure 2:**
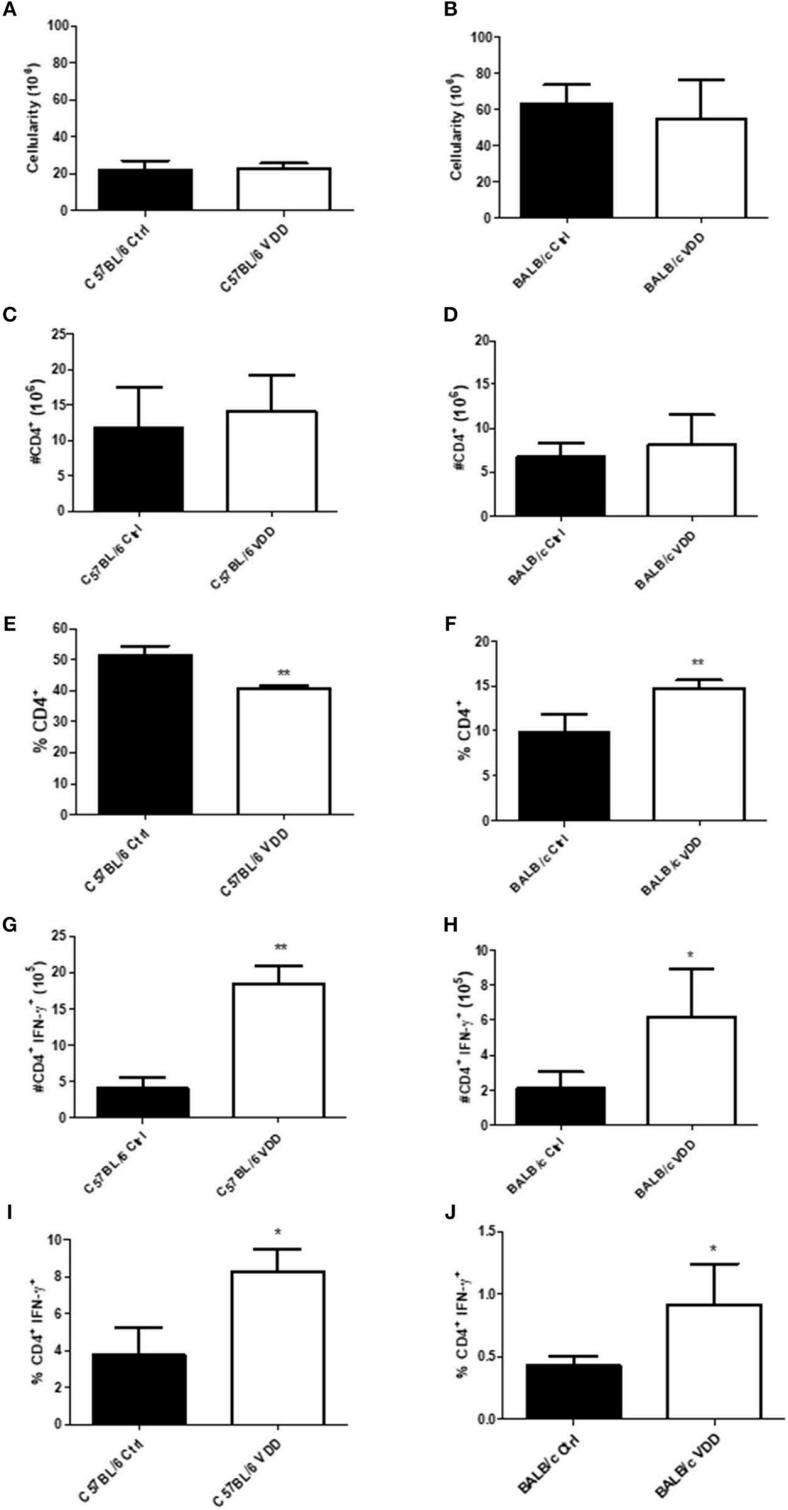
Increase in the T CD4^+^ IFN-γ^+^ cell population in lesion-draining lymph nodes of *L. (L.) amazonensis*-infected VDD mice. C57BL/6 and BALB/c mice normally fed (Ctrl) or on a Vitamin D-deficient diet (VDD) were subcutaneously infected in the footpad with 2 × 10^5^ *L. (L.) amazonensis* promastigotes. On day 92 (C57BL/6) or 99 (BALB/c) post-infection, lesion-draining lymph node cellularity **(A,B)**, number of T CD4^+^ lymphocytes **(C,D)**, percentage of T CD4^+^ lymphocytes **(E,F)**, number of IFN-γ producing T CD4^+^ lymphocytes **(G,H)** and percentage of IFN-γ producing T CD4^+^ lymphocytes **(I,J)** were assessed by flow cytometry. The data (means ± SD; *n* = 5 ^**^P < 0.0001) are representative of two independent experiments producing the same result profile.

To better understand the role of VitD in the immune response to infection, cell phenotype in lesion-draining popliteal lymph nodes was evaluated at the endpoint of infection, day 92 for C57BL/6, and day 99 for BALB/c. There was no difference in the total number of lymph node cells between VDD and Ctrl mice, in both mouse strains, C57BL/6 (Figure 2A) and BALB/c (Figure 2B). There was also no difference in the number of CD4^+^ cells between VDD and Ctrl mice (Figures 2C,D), but in terms of the percentage of CD4^+^ cells in the total cell population, there was a reduction in C57BL/6 VDD (Figure 2E) and an increase in BALB/c VDD (Figure 2F) compared to their respective controls. Since *Leishmania* spp. are intracellular parasites and induction of Th1 response is generally associated with disease control, the number and percentage of the CD4^+^ cell population producing IFN-γ was further evaluated. The CD4^+^ IFN-γ^+^ cell population was increased in both absolute number (Figures 2G,H) and the percentage within the CD4^+^ population (Figures 2I,J) in C57BL/6 VDD and BALB/c VDD mice compared to respective controls. This cell population may be associated with resistance of mice to *L. (L.) amazonensis* infection.

### BALB/c VDD Exhibit a Reduction in IL-10 and IgE Levels, Without Affecting IgG1 and IL-4 Production

**Figure 3:**
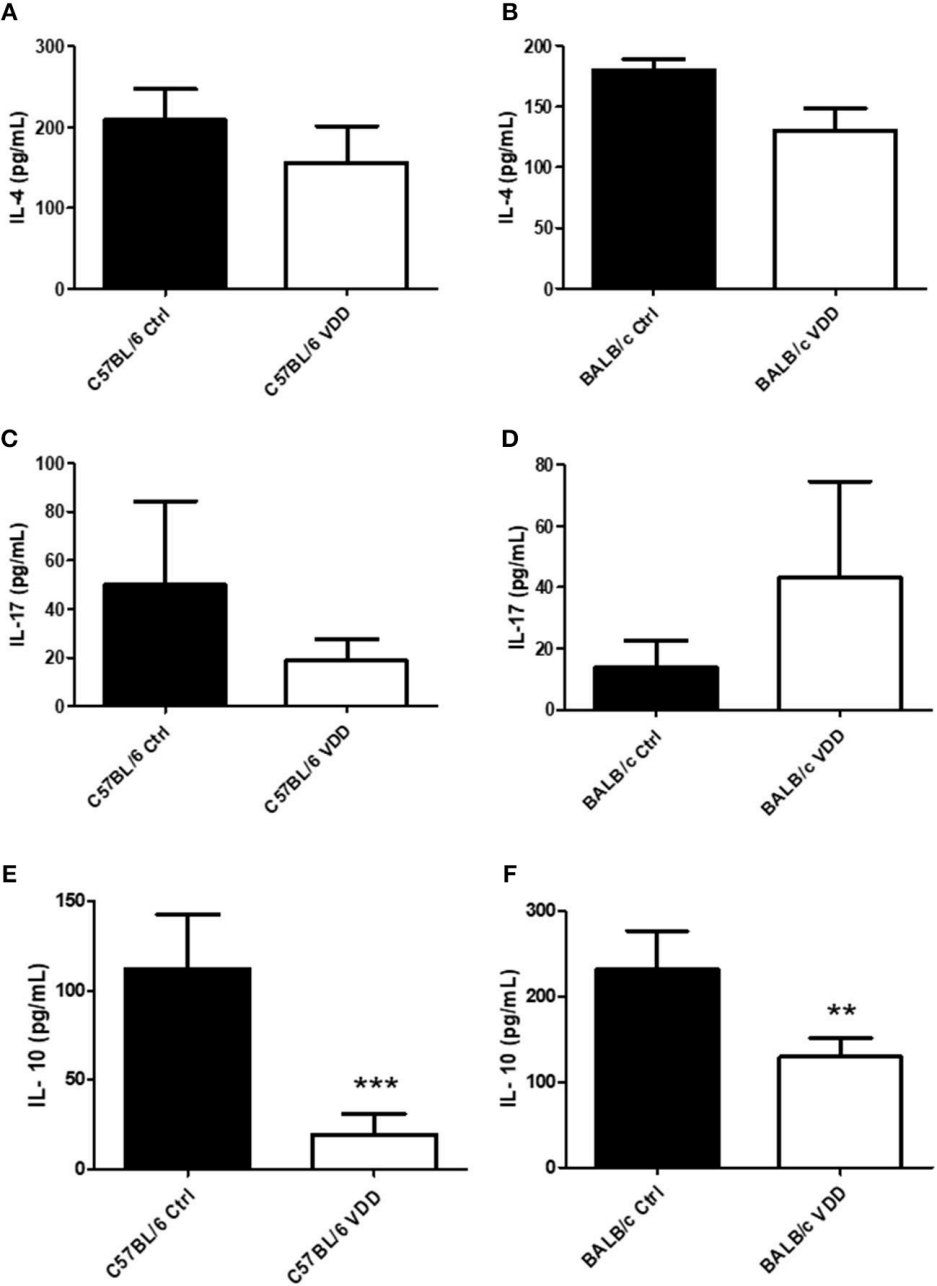
Cytokine profile in the lesions of *L. (L.) amazonensis*-infected VDD mice. C57BL/6 and BALB/c mice normally fed (Ctrl) or on a Vitamin D-deficient diet (VDD) were subcutaneously infected in the footpad with 2 × 10^5^ *L. (L.) amazonensis* promastigotes. On day 92 (C57BL/6) or 99 (BALB/c) post-infection, the cytokine profile in the lesions was evaluated by ELISA. IL-4 **(A,B)**, IL-17 **(C,D)**, and IL-10 **(E,F)** levels in tissue homogenates were quantified. The data (means ± SD; *n* = 5; ^***^P < 0.001, ^**^P < 0.01) are representative of two independent experiments producing the same result profile.

**Figure 4:**
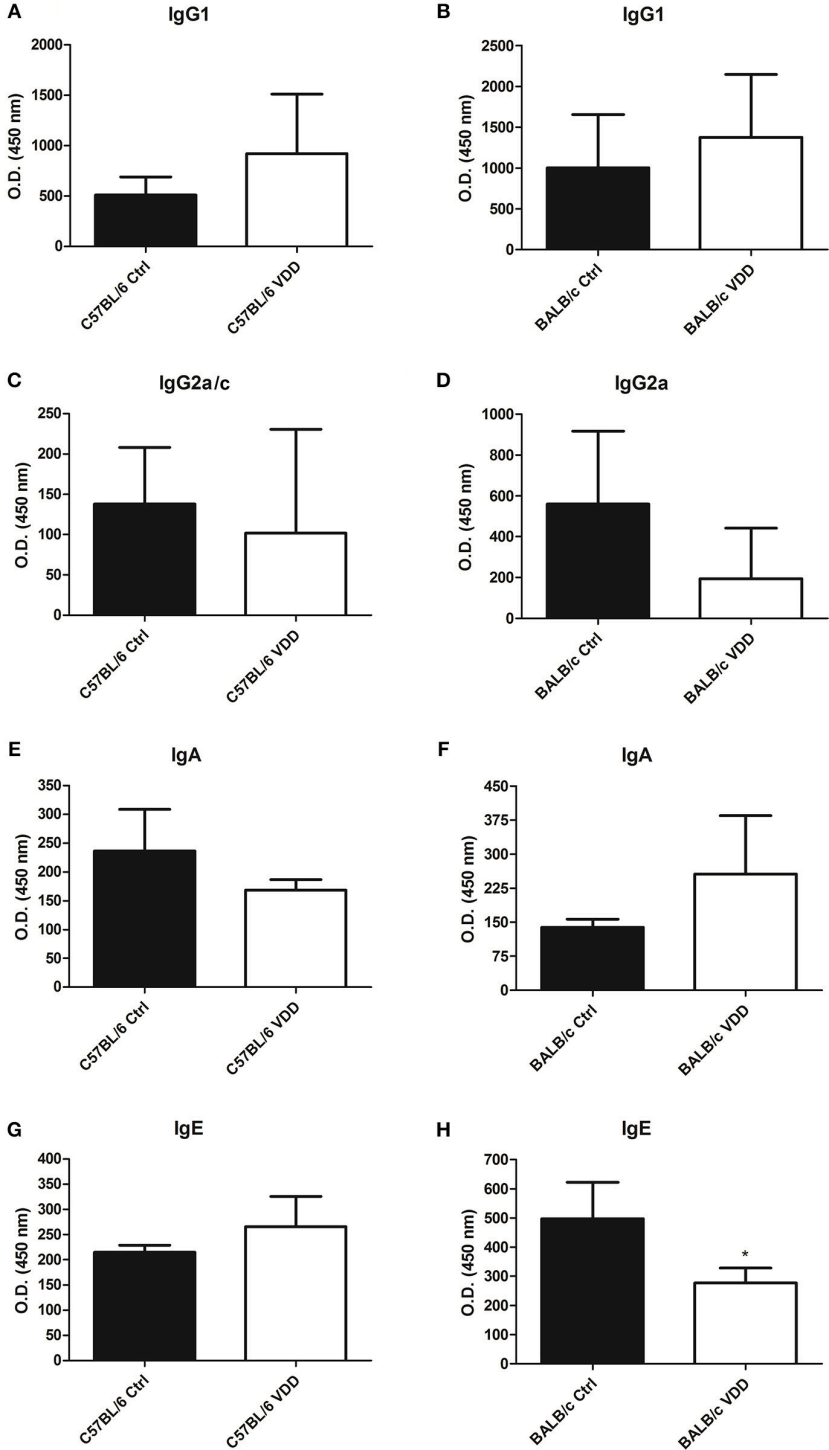
*L. (L.) amazonensis*-specific antibody production in VDD mice. C57BL/6 and BALB/c mice normally fed (Ctrl) or on a Vitamin D-deficient diet (VDD) were subcutaneously infected in the footpad with 2 × 10^5^ *L. (L.) amazonensis* promastigotes. On day 92 (C57BL/6) or 99 (BALB/c) post-infection, *L. (L.) amazonensis*-specific antibody production in the serum was evaluated by ELISA. IgG1 **(A,B)**, IgG2a **(C,D)**, IgA **(E,F)**, and IgE **(G,H)** levels were quantified. Mean ± SD; *n* = 5. The data (means ± SD; *n* = 5; ^*^P < 0.05) are representative of two independent experiments producing the same result profile.

Cytokine and antibody production at the endpoint of infection, day 92 for C57BL/6 and day 99 for BALB/c, were also evaluated. Both C57BL/6 VDD and BALB/c VDD mice showed no difference in IL-4 and IL-17 production compared to respective controls (Figures 3A–D), however, a decrease of IL-10 was observed in VDD mice in comparison with Ctrl mice (Figures 3E,F). Moreover, both C57BL6 VDD and BALB/c VDD mice had no difference in the production of IgG1, IgG2a, and IgA antibodies specific for *L. (L.) amazonensis* antigens compared to respective controls (Figures 4A–F). However, BALB/c VDD mice showed reduced levels of IgE compared to the control mice on the regular diet (Figure 4H).

## Discussion

Since the leishmaniases are neglected diseases that mainly affect poor populations, dietary factors could likely influence the development of infection. The hypothesis of a possible VitD involvement in the regulation of immune processes was due to the presence of an abundance of VDR receptors in different cell types of the immune system, such as CD4^+^ and CD8^+^ T lymphocytes, neutrophils, dendritic cells, and macrophages (Baeke et al., 2010). In addition, VitD plays a role in the development of Th2 and Treg immune responses, and in the induction of lymphocyte expression of molecules that target homing of cells to the skin (Sassi et al., 2018).

VDD mice exhibited a smaller lesion progression profile, although they did not present a significant reduction in the parasite load, compared to control mice fed on a regular diet (Figure 1). This profile was observed in both partially susceptible C57BL/6 and susceptible BALB/c mice. A previous study described that C57BL/6 VitD-receptor knockout (VDRKO) mice are also more resistant to *L. (L.) major* infection compared to wild-type (WT) mice (Ehrchen et al., 2007; Whitcomb et al., 2012). However, BALB/c VDRKO mice present no difference in lesion development during *L. (L.) major* infection compared WT mice (Whitcomb et al., 2012). Together, these data indicate that VDD, either through dietary VitD depletion or the ablation of VitD-signaling, leads to an increased resistance to *Leishmania* infection. It is important to note that experimental models lacking VDR expression are quite different from the dietary VitD deprivation model used in this study. VDRKO animals have an absence of a systemic response to VitD, affecting cell differentiation and impacting on the innate and adaptive immune system. On the other hand, the use of a deficient diet does not impair the endogenous synthesis of VitD or its signaling pathway, being the best model to study the nutritional impact. Our data show that using VDD mice, either in a C57BL/6 or BALB/c background, a similar resistance to *L. (L.) amazonensis* infection was observed, indicating a strong phenotype using the VitD deficient diet.

Contrary to what was observed in CL, VitD deficiency is related to the progression of VL in dogs (Rodriguez-Cortes et al., 2017), suggesting clear differences in the involvement of macrophages from skin and the visceral organs, such as spleen, liver, and bone marrow. Clinical studies in humans should be performed to better comprehend the impact of VitD deficiency in CL and VL.

A different approach showed that intraperitoneal treatment with the active form of VitD, 1,25-dihydroxycholecalciferol [1,25(OH)_2_D_3_] significantly reduces lesion size in BALB/c mice infected with *L. (L.) mexicana*, but without reducing the parasite load. Treatment with 1,25(OH)_2_D_3_ is associated with a healing profile, increasing eosinophils and fibroblasts in the lesions, enhancing collagen production, and decreasing pro-inflammatory cytokines levels compared to untreated control mice, without affecting parasite elimination (Ramos-Martínez et al., 2013). However, this model uses the active form of VitD after infection, while the VitD deficient diet affects immunity in the mice prior to infection. Thus, it is not adequate to compare results between these different approaches, and dietary VitD deficiency is the most appropriate model to evaluate the impact of nutrition on immunity against leishmaniasis.

Comparing C57BL/6 ctrl mice (partially resistant) to BALB/c ctrl mice (susceptible), C57BL/6 mice exhibited higher numbers of IFN-γ-producing CD4^+^ cells, which are related to leishmaniasis control (Figure 2). It has been demonstrated that VitD suppresses the Th1 response (Cantorna et al., 2015). Corroborating this data, our results show that both C57BL/6 and BALB/c VDD have an increased frequency and number of IFN-γ-producing CD4^+^ cells (Figure 2). This increased Th1 response is probably related to the more controlled lesion development in infected VDD mice. However, the increase of IFN-γ-producing cells was not enough to control the parasite load, so perhaps other mechanisms are necessary, such as reactive oxygen species production by macrophages. Whereas, VDRKO mice also exhibit an increase in IFN-γ production by CD4^+^ and CD8^+^ T cells during *L. (L.) major* infection, this controls both lesion development and parasite load (Ehrchen et al., 2007). In our experiments, no difference in CD8^+^ T cells was observed (data not shown), which may be associated to the failure to control parasite load.

Evaluation of cytokine production at the site of infection in the chronic phase showed that VDD mice, both C57BL/6, and BALB/c, have no difference in IL-4 (Figure 3), which has also been observed in *L. (L.) major* infection of C57BL/6 VDRKO mice (Whitcomb et al., 2012). *In vitro* studies indicate that VitD upregulates the activity of Th2 cells (Boonstra et al., 2001; Staeva-Vieira and Freedman, 2002). IL-4 is a cytokine associated with susceptibility to *L. (L.) amazonensis* infection and IL-4-deficient BALB/c mice are more resistant to infection, depending on the initial parasite inoculum, developing smaller lesions and a higher Th1 response, with higher IFN-γ, and IgG2a production than WT mice (Guimarães et al., 2006). In addition, leishmaniasis pathogenesis is associated with the blockage of a Th1 response development, leading to a greater number of IL-4 and IL-10-producing cells in the lesions (Carvalho et al., 2016). Studies with C57BL/6 mice show that only 30% of IL-4-receptor-deficient animals develop a lesion after inoculation of *L. (L.) amazonensis* and they have higher IFN-γ production compared to WT mice (Felizardo et al., 2012). Despite the fact that VitD is related to a Th2 immune response development, dietary VitD deficiency did not impact IL-4 production during *L. (L.) amazonensis* infection. It has also been demonstrated that VitD suppresses Th17 cell differentiation (Korf et al., 2012; Fawaz et al., 2016), a cytokine related to pathogenesis of *L. (L.) mexicana* infection (Pedraza-Zamora et al., 2017). However, no difference in the level of IL-17 was observed between VDD and Ctrl mice.

We demonstrated that VDD mice presented a reduction of IL-10 in comparison to Ctrl mice (Figures 3E,F). A similar phenotype was observed in C57BL6 VDRKO mice, with a decrease of IL-10 by Real Time PCR in comparison to WT mice (Whitcomb et al., 2012). IL-10 production has been associated to susceptibility to *L. (L) amazonensis* infection (Padigel et al., 2003) and reduction of IL-10 is related to a decrease of lesion size (Padigel et al., 2003; Firmino-Cruz et al., 2018). In addition, the production of IFN-γ inhibits the production of IL-10 by macrophages (Herrero et al., 2003). Therefore, we suggest VitD deficiency promotes the increase of IFN-γ that inhibits the production of IL-10, which is associated with reduction of lesion size.

Antibody production has been associated to pathogenesis in *L. (L.) amazonensis* infection (Firmino-Cruz et al., 2018). Our results show that VDD mice had no difference in antigen specific-IgG1 and IgG2a production compared to Ctrl mice (Figure 4). However, it has been shown that C57BL/6 VDRKO mice infected with *L. (L.) major* produce more IgG2a in comparison to WT mice (Whitcomb et al., 2012). With regards to BALB/c VDD mice, there was a higher serum level of IgE compared to Ctrl mice, which is related to a Th2 immune response. In humans, VitD deficiency is related to increased IgE with direct effect on B cells during allergy (James et al., 2016; Guo et al., 2018).

Studies using experimental models have demonstrated that lack of VitD also interferes with the immune response to other pathogens. VDD mice have increased susceptibility to lung infection by *Aspergillus fumigatus*, developing an excessive inflammatory response (Li et al., 2014). VitD also plays a key role in the immunity against *Mycobacterium tuberculosis* in mice. Following BCG stimulation, IFN-γ levels significantly increased and IL-10 levels significantly decreased in the VDD mice compared to control mice (Yang et al., 2013). On the other hand, 1,25(OH)_2_D_3_ treatment inhibited the inflammatory infiltrates and expression of IL-2, IFN-γ and TNF-β in the spleen of VDD mice following vaccination with BCG (Zhang et al., 2018). These findings corroborate that Th1 cells are strongly activated under VDD conditions.

## Conclusion

Altogether, our results indicate that dietary VitD deficiency is able to decrease lesion growth and provide an increase in Th1 response in C57BL/6 and BALB/c mice upon *L. (L.) amazonensis* infection, although it does not decrease parasite burden in either of the murine models used. Thus, VitD may contribute to host susceptibility to murine tegumentary leishmaniasis. Further studies on the influence of immunonutrition in the leishmaniases are needed to better understand the immunobiology of these diseases.

## Author Contributions

HdMG conceived and designed the experiments. GO-S, DB, MM, JEP, JCP, TR, AdF-M, and LF-C performed the experiments. GO-S, MM, LF-C, TR, and AdF-M analyzed data. IB, GO-S, DG, BR-B, and HdMG scientific discussion. HdMG, BR-B, and AV contributed reagents, materials and analysis tools. IB and HdMG wrote the paper.

### Conflict of Interest Statement

The authors declare that the research was conducted in the absence of any commercial or financial relationships that could be construed as a potential conflict of interest.

## Supplementary Material

Supplementary Figure 1 Parasite loads in draining lymph nodes. C57BL/6 and BALB/c mice normally fed (Ctrl) or on a Vitamin D-deficient diet (VDD) were subcutaneously infected in the footpad with 2 × 10^5^ *L. (L.) amazonensis* promastigotes and lesion development was followed weekly as in Figure 1. On day 92 (C57BL/6) or 99 (BALB/c) post-infection, parasite loads in the draining lymph nodes were evaluated by limiting dilution assay **(A,B)**. The data are representative of three independent experiments producing the same result profile.

Supplementary Figure 2 Body weight variation upon *L. (L.) amazonensis* infection. C57BL/6 **(A)** and BALB/c **(B)** mice fed normally (Ctrl) or on a Vitamin D-deficient diet (VDD) were subcutaneously infected in the footpad with 2 × 10^5^ *L. (L.) amazonensis* promastigotes 45 days after starting the diet. Body weight was evaluated weekly throughout the experiment. The data (means ± SD; *n* = 5) are representative of three independent experiments producing the same result profile.

Supplementary Figure 3 Evaluation of VDD mice on high challenge model of infection. C57BL/6 and BALB/c mice normally fed (Ctrl) or on a Vitamin D-deficient diet (VDD) were subcutaneously infected in the footpad with 2 × 10^6^ *L. (L.) amazonensis* promastigotes and lesion development was followed weekly **(A,B)**. The data (means ± SD; *n* = 5) are representative of two independent experiments producing the same result profile.

Supplementary Figure 4 C57BL/6 VDD mice are more resistant to *L. (L.) amazonensis* infection in late chronic phase. C57BL/6 normally fed (Ctrl) or on a Vitamin D-deficient diet (VDD) were subcutaneously infected in the footpad with 2 × 10^5^ *L. (L.) amazonensis* promastigotes and lesion development was followed weekly **(A)**. On day 149 post-infection, parasite loads in the infection site were evaluated by limiting dilution assay **(B)**. The data (means ± SD; *n* = 5 ^**^*P* < 0.001; ^*^*P* < 0.05) are representative of two independent experiments producing the same result profile.

Supplementary Figure 5 Gate strategy used for lymph node CD4^+^ cells. Lymph node cells from infected Ctrl and VDD mice were plated at 5 × 10^5^ per well and stained for flow cytometry to determine the percentage of CD4^+^ and CD4^+^ IFN-γ^+^ cells.
