# Supplementary figures and images for: Dietary Vitamin D3 Deficiency Increases Resistance to Leishmania (Leishmania) amazonensis Infection in Mice
Source: Front Cell Infect Microbiol. 2019 Apr 9;9:88. doi: 10.3389/fcimb.2019.00088 (PMC6467002; doi:10.3389/fcimb.2019.00088)

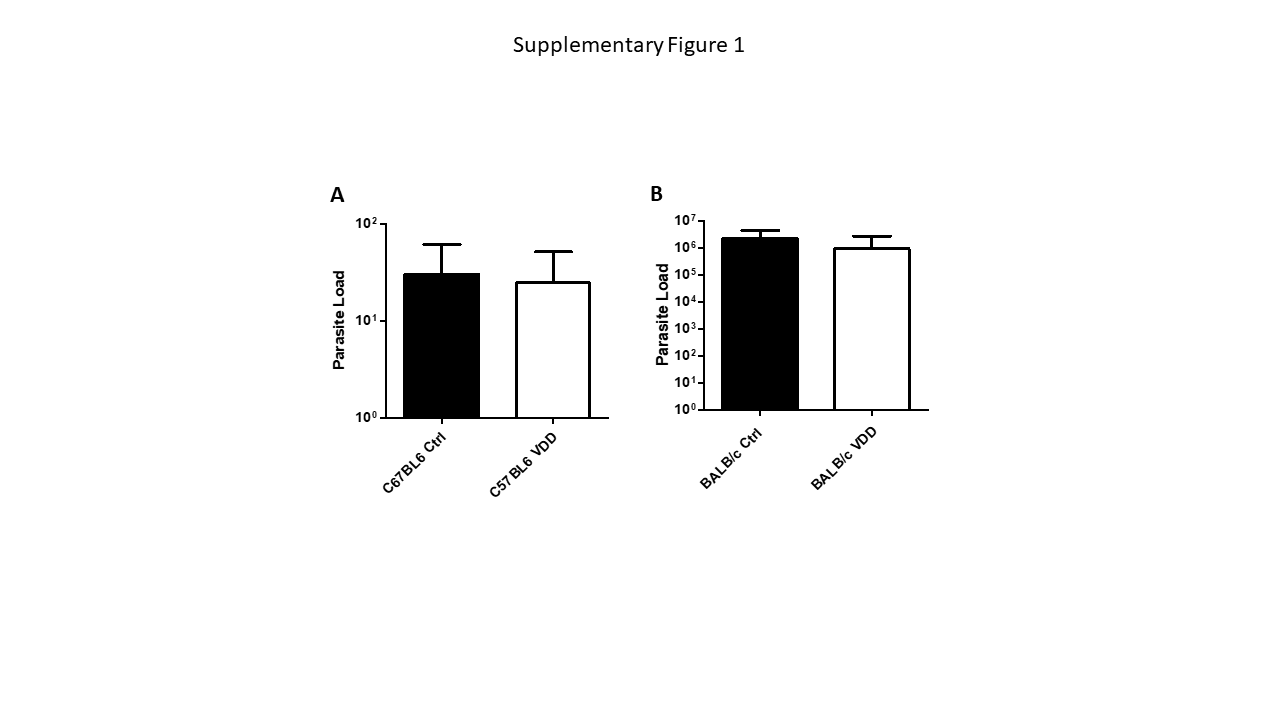

Supplement: Supplementary Figure 1 — Parasite loads in draining lymph nodes. C57BL/6 and BALB/c mice normally fed (Ctrl) or on a Vitamin D-deficient diet (VDD) were subcutaneously infected in the footpad with 2 × 10^5^ L. (L.) amazonensis promastigotes and lesion development was followed weekly as in Figure 1. On day 92 (C57BL/6) or 99 (BALB/c) post-infection, parasite loads in the draining lymph nodes were evaluated by limiting dilution assay (A,B). The data are representative of three independent experiments producing the same result profile. [file Image_1.TIF]

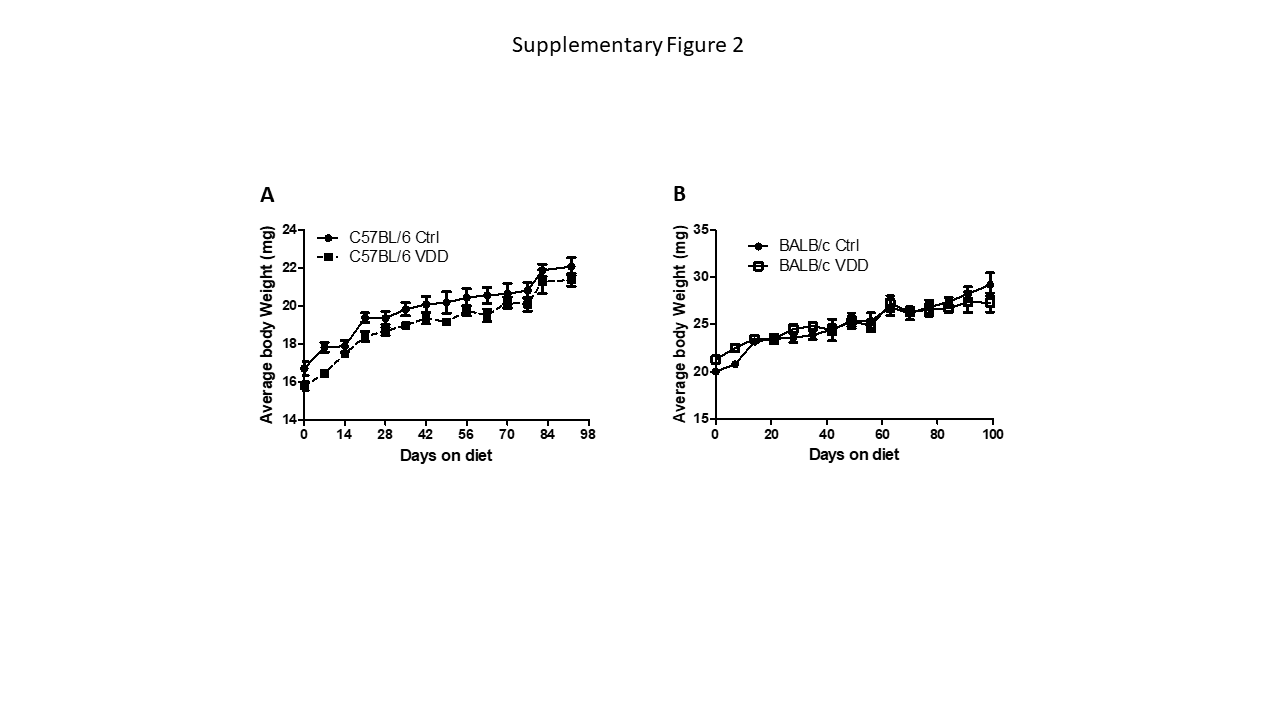

Supplement: Supplementary Figure 2 — Body weight variation upon L. (L.) amazonensis infection. C57BL/6 (A) and BALB/c (B) mice fed normally (Ctrl) or on a Vitamin D-deficient diet (VDD) were subcutaneously infected in the footpad with 2 × 10^5^ L. (L.) amazonensis promastigotes 45 days after starting the diet. Body weight was evaluated weekly throughout the experiment. The data (means ± SD; n = 5) are representative of three independent experiments producing the same result profile. [file Image_2.TIF]

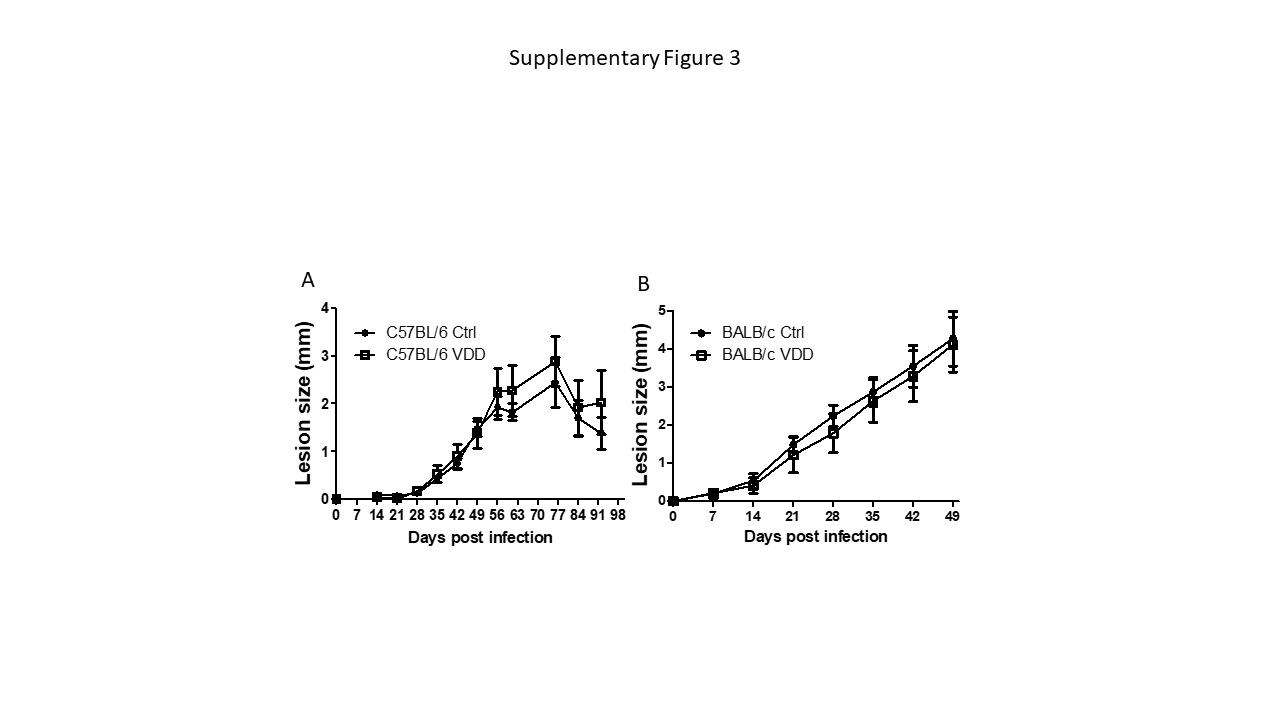

Supplement: Supplementary Figure 3 — Evaluation of VDD mice on high challenge model of infection. C57BL/6 and BALB/c mice normally fed (Ctrl) or on a Vitamin D-deficient diet (VDD) were subcutaneously infected in the footpad with 2 × 10^6^ L. (L.) amazonensis promastigotes and lesion development was followed weekly (A,B). The data (means ± SD; n = 5) are representative of two independent experiments producing the same result profile. [file Image_3.TIF]

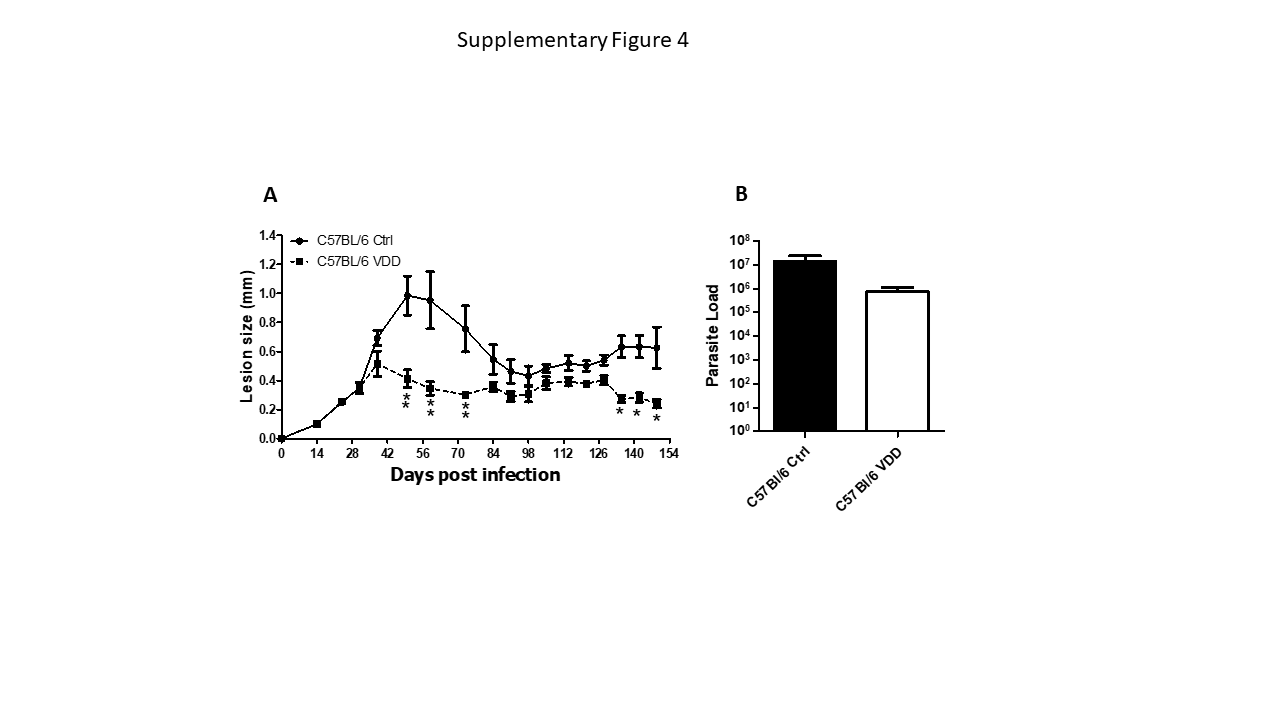

Supplement: Supplementary Figure 4 — C57BL/6 VDD mice are more resistant to L. (L.) amazonensis infection in late chronic phase. C57BL/6 normally fed (Ctrl) or on a Vitamin D-deficient diet (VDD) were subcutaneously infected in the footpad with 2 × 10^5^ L. (L.) amazonensis promastigotes and lesion development was followed weekly (A). On day 149 post-infection, parasite loads in the infection site were evaluated by limiting dilution assay (B). The data (means ± SD; n = 5 **P < 0.001; *P < 0.05) are representative of two independent experiments producing the same result profile. [file Image_4.TIF]

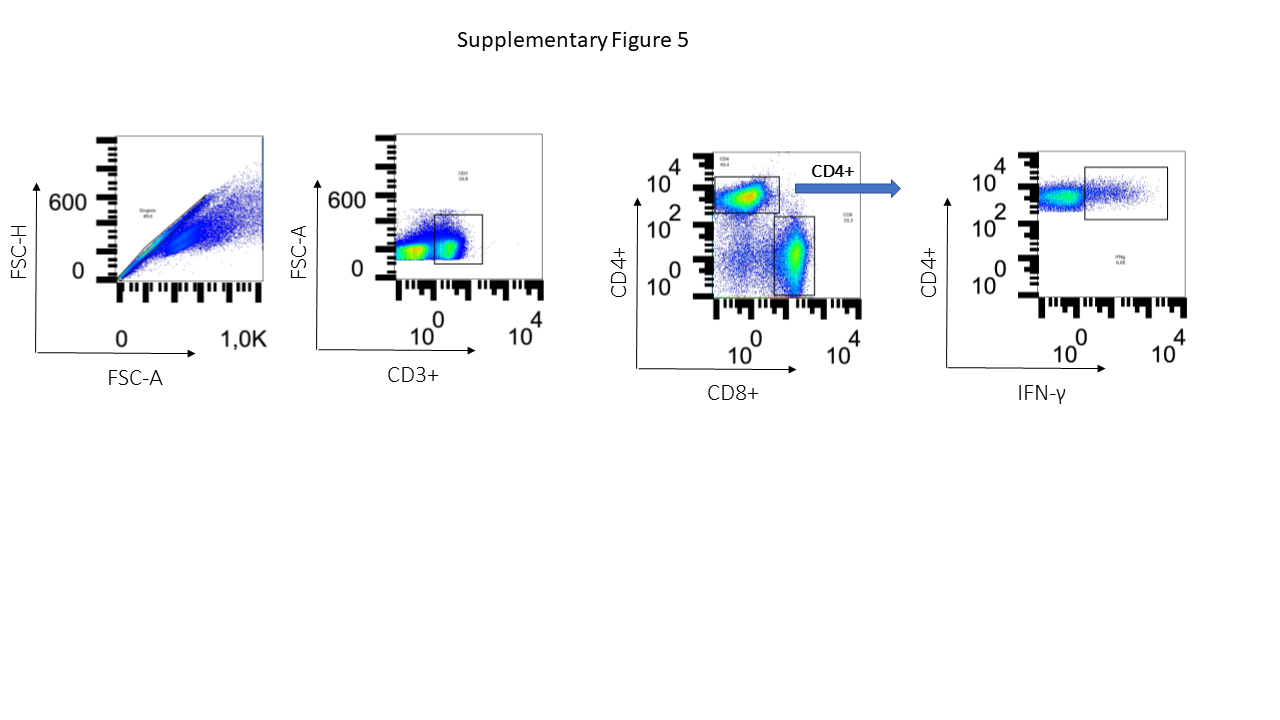

Supplement: Supplementary Figure 5 — Gate strategy used for lymph node CD4+ cells. Lymph node cells from infected Ctrl and VDD mice were plated at 5 × 10^5^ per well and stained for flow cytometry to determine the percentage of CD4+ and CD4+ IFN-γ+ cells. [file Image_5.TIF]
